# Cultural determinants and resilience and recovery factors associated with trauma among Aboriginal help-seeking clients from an Aboriginal community-controlled counselling service

**DOI:** 10.1186/s12888-023-04567-5

**Published:** 2023-03-10

**Authors:** Graham Gee, Carol Hulbert, Helen Kennedy, Yin Paradies

**Affiliations:** 1 Intergenerational Health Group, Murdoch Children’s Research Institute, Intergenerational Health Group, Royal Children’s Hospital, Level 5, 50 Flemington Road, Parkville, VIC 3052 Australia; 2 School of Psychological Sciences, University of Melbourne, Parkville, VIC Australia; 3 Victorian Aboriginal Health Service, (2008-2018), Fitzroy, VIC Australia; 4 Victorian Aboriginal Health Service, (2008-2012), Fitzroy, VIC Australia; 5 Department of Paediatrics, University of Melbourne, Parkville, VIC Australia; 6 School of Humanities and Social Science, Faculty of Arts and Education, Deakin University, Burwood, VIC Australia

**Keywords:** Aboriginal mental health, Social and emotional wellbeing, Cultural determinants or wellbeing, Trauma, Resilience, Recovery

## Abstract

In addition to resilience and resistance, collective and personal experiences of trauma are commonly cited within the context of Aboriginal and Torres Strait Islander and other Indigenous First People’s experiences of colonisation. This study investigated whether a range of risk and protective factors, including cultural determinants of social and emotional wellbeing, were associated with posttraumatic stress outcomes among 81 Aboriginal help-seeking clients from an Aboriginal community-controlled counselling service in Melbourne, Australia. The study explored potential relationships between trauma exposure, child removal from natural family, experiences of racism, gender, and trauma symptom severity. The study also investigated whether personal, relationship, community and cultural strengths and determinants of wellbeing, as detailed in the Aboriginal Resilience and Recovery Questionnaire, moderated the relationship between trauma exposure and posttraumatic stress symptom severity. Participants commonly endorsed symptoms of distress consistent with Posttraumatic Stress Disorder and cultural idioms of distress as documented in the Aboriginal Australian Version of the Harvard Trauma Questionnaire. Two generations of child removal from one’s natural family, experiences of racism, stressful life events experienced during the past 12 months, being male, and not having access to funds for basic living expenses were all associated with greater trauma symptom severity. Conversely, participants self-reported access to personal, relationship, community and cultural strengths was associated with lower trauma symptom severity. Regression analysis revealed that trauma exposure, stressful life events, access to basic living expenses, and personal, relationship, community, and cultural strengths were all important predictors of posttraumatic stress symptom severity. Participant access to strength and resources that included connections to community and culture, moderated the relationship between trauma exposure and trauma symptom severity.

## Introduction

Human inflicted violence and its effects on individuals, communities and nations worldwide have been documented throughout history, occurring in every culture and across all nationalities and races of people. Intergenerational trauma and collective violence are often cited within the context of Indigenous^1^ people’s historical experiences of colonisation [1–3], while elevated rates of interpersonal violence have been the subject of governmental and Aboriginal and Torres Strait Islander^1^ and other Indigenous-led reports [4–7]. Both forms of violence hold significance for the *Koori*^2^First Peoples of Victoria in South Eastern Australia. Historical records [8], ethnographic research [9], and the recorded stories of Koori people themselves [10, 11], show that colonisation for the Koori clan groups of Victoria involved systemic structural violence and oppression, the impacts of which are consistent with large scale collective trauma. Documentation of the rates of interpersonal violence among Aboriginal Victorians is also readily accessible, with data from health surveys indicating higher rates of hospitalisation for assault and intentional self-harm, and a greater likelihood of being a victim of threatened physical violence in the last 12 months, in comparison to non-Aboriginal Victorians [12]. Concurrently, other Victorian Aboriginal population health statistics indicate significant disparities across multiple social determinants of health in comparison to other Victorians, including lower income and educational attainment, and higher rates of unemployment and financial distress [12, 13].

These current health inequalities and experiences of past adversity suggest that Koori and other Aboriginal Victorians may be particularly vulnerable to experiencing the effects of trauma. This is not to suggest that all Aboriginal Victorians are victims of traumatisation. Rather, that a proportion of Aboriginal Victorians may be at a higher risk of experiencing the effects of trauma, specifically those who may have experienced intergenerational, multiple adversities, such as the cumulative effects of historical and cultural loss, high levels of exposure to violence and/or other stressful life events, and inequalities across multiple social determinants. However, mental health outcomes associated with past and contemporary potentially traumatic events among Aboriginal Victorians remains relatively unexplored. For instance, the prevalence rate of Posttraumatic Stress Disorder (PTSD) among Aboriginal Victorians is not yet known. Although there are diverging views about the adequacy and applicability of the PTSD construct in the Indigenous literature, as a trauma concept it has also been utilized by Indigenous peoples worldwide [14, 15]. Large to medium scale surveys and studies involving Indigenous populations conducted in the United States, New Zealand and Australia indicate that PTSD rates appear to be approximately one and a half to two times higher than that of non-Indigenous populations [16–18]. One limitation of these large population studies is that they examined PTSD symptom criterion only (e.g., re-experiencing, avoidance, hyper-arousal), and did not include cultural idioms of trauma-related distress that may be significant for Aboriginal and Torres Strait Islander people [19, 20].

Currently, research investigating associations between strengths and protective factors, and posttraumatic stress symptom severity within Indigenous mental health contexts appears scarce. However, other sources of national and international literature that explore healing and trauma recovery within Indigenous contexts are highly relevant. One is the multiple reports stemming from key inquiries that involved consultation with Aboriginal and Torres Strait Islander communities about how to address past and contemporary experiences of trauma. A common theme in these reports is that the magnitude of trauma experienced by some Aboriginal and Torres Strait Islander communities requires greater investment in service delivery and coordination; capacity building in community level programs that target prevention and healing of trauma; strengthening community control and self-determination; and overcoming of social and economic disadvantage, racism, and dispossession from land and culture [7, 21, 22]. One implication of these recommendations is that system and structural level resources are a necessary pre-requisite for creating environments where healing and recovery from large-scale trauma is possible [23, 24].

Only a handful of studies involving Aboriginal and Torres Strait Islander participants have systematically examined healing and recovery from trauma. J. Atkinson (2002) conducted qualitative interviews with six Aboriginal adults participating in the We Al-li program and identified nine themes that described their experiences of healing from trauma. These themes included healing as an awakening experience; healing as an experience of safety; healing as community support; rebuilding a sense of family and community; increasing self-knowledge; ceremony as healing; culture and spirituality in healing; healing as experiences of transformation and transcendence; and healing as integration. This occurred in processes where participants were able tell their story and find meaning, experience emotions associated with their histories of trauma, work through loss and grief to find acceptance, and for some, reclaim a sacred sense of self [25]. Gilmour independently evaluated 21 healing projects funded by the Aboriginal and Torres Strait Islander Healing Foundation from 2011 to 2013 and found similar ‘drivers of a quality healing program’, including that healing practices were derived from local culture and values; provided a safe place for healing to occur; targeted the impact of colonisation and trans-generational trauma and grief; addressed specific local community issues and were driven by local leadership; were proactive rather than reactive in building capacity; provided local mentoring; involved Elders and incorporated spirituality; applied principles of social justice and human rights; and tended to use a blend of Aboriginal healing and western practices ([26] p. 20).

Arguably, the largest evidence base documenting Aboriginal people’s experiences of healing and recovery from trauma comes from the First Nations Canadian Aboriginal Healing Foundation (Healing Foundation). During its first seven years, the Healing Foundation funded nearly 1400 Aboriginal community-driven and designed healing projects, and in its evaluation processes interviewed over 100 organisations, and more than 1,400 participants. Among many findings, the Healing Foundation identified ‘three pillars of healing’ common to many programs, which included reclaiming history, cultural interventions, and therapeutic healing. Situated below these pillars were a series of personal, family and community history related factors that varied considerably between individuals, which the Healing Foundation suggested significantly impacted upon a person’s need for healing, and the success or failure of the healing process. The Healing Foundation reported that it was unable to conduct research at the level of individual differences, and among recommendations was the need for community-driven research that investigated individual differences in these healing and recovery variables [27, 28].

One of the strengths of existing reports and studies examining Indigenous peoples’ experiences of healing and recovery from trauma is that many describe similar phases of healing and similar strengths and common practices, suggesting a degree of reliability regarding observations of important aspects of healing and recovery from trauma. A limitation of current research is that there is no reliable data documenting individual differences with regards to the types of strengths and practices cited in the literature. Hence, the relationship between commonly cited strengths (i.e., potential moderating factors) and post trauma outcomes remains unclear. At a therapeutic level, this manifests as a lack of knowledge with regards to fully understanding why it is that some Aboriginal help-seeking clients who attend counselling experience poorer post-trauma outcomes than others. Post-trauma outcomes could be a function of utilising more or less strengths, experiencing more or less trauma, accessing more or less resources, and experiencing higher or lower levels of stress and social adversity. The Posttraumatic Stress Disorder (PTSD) literature has clearly established the role of prior trauma exposure and post-trauma stressful life events in contributing to posttraumatic stress symptom severity [29]. An important question is how do experiences of trauma and stressful life events interact with strengths and protective factors, including cultural determinants of wellbeing, to contribute to trauma related outcomes among Aboriginal and Torres Strait Islander help-seeking peoples? This question concerned counselors and Aboriginal health workers alike at the Family Counseling Services (FCS) unit of the Victorian Aboriginal Health Service, in Melbourne Australia, where many Koori and Victorian Aboriginal people seek help for their social and emotional wellbeing and mental health needs.

## Present study

The study and findings reported in this article were part of a larger PhD program undertaken by the first author that utilised a mixed-method research design to examine historical loss, contemporary trauma, and resilience and recovery outcomes among Aboriginal help-seeking clients from the FCS. Using the Aboriginal Resilience and Recovery Questionnaire developed at the service [30] this study reports on findings from conducting structured interviews with clients to investigate individual differences in strengths, and potential interactions between a wide range of individual, social and cultural factors that may have contributed to post trauma outcomes. Based on qualitative findings from focus groups with Aboriginal health professional working at the FCS [30], the first aim of the study was to investigate potential relationships that might exist between trauma symptom severity, and child removal from natural family, experiences of racism and being male. The second aim was to investigate potential protective effects of resilience in relation to trauma symptom severity—specifically the strengths and resources accessed by clients as detailed in the ARRQ.

## Method

The study was reviewed and approved by the University of Melbourne Behavioral and Social Sciences Human Ethics Subcommittee, the University of Melbourne Human Research Ethics Committee, and the Victorian Aboriginal Health Service research subcommittee and Board of Directors in 2010. The methodology of the study and broader PhD program was grounded in Aboriginal scholar Lester-Irabinna Rigney’s three interrelated research principles that included privileging Aboriginal voices, political integrity, and resistance as the emancipatory imperative [31, 32]. It also utilised Maori researcher Sir Mason Durie’s concept of research at the interface of Indigenous knowledge and science [33], with the design of the study also located in the field of psychology and public health.

Independent t-tests and pearson correlations were conducted to examine our hypotheses that: (1) client’s with personal experiences of child removal from natural family would report greater posttraumatic stress symptom severity in comparison to those who did not experience removal; (2) experiences of racism would be associated with greater posttraumatic stress symptom severity; and, (3) contrary to consistent findings in the worldwide PTSD literature, male Aboriginal help-seeking clients would report greater trauma symptom severity than female Aboriginal help-seeking clients. An exploratory analysis was also conducted to examine associations between trauma symptom severity and a range of strengths and cultural determinants of wellbeing from the subscales of the ARRQ (Gee, 2016), including: cultural identity, cultural practices, spirituality, community connection, opportunities in community, communal mastery, and safety. Hierarchical regression analysis was used to examine our hypothesis that (4) total strength scores would moderate the relationship between trauma exposure and trauma symptom severity, after controlling for financial distress. We also conducted an exploratory hierarchical regression analysis to investigate the contributing role of trauma exposure, stressful life events, and resilience in predicting trauma symptom severity, after controlling for financial distress.

## Participants

Structured interviews were conducted with 81 Aboriginal help-seeking clients who used the FCS during 2011. Sixty per cent of participants (*n* = 48) were attending the FCS on a monthly basis and receiving regular therapeutic support from a general medical practitioner, psychiatrist, counsellor, psychologist, drug and alcohol worker, or the service’s Aboriginal men’s and women’s group. The other 40 per cent (*n* = 33) were a diverse group of clients and community members. These participants regularly accessed services at the main site of the Victorian Aboriginal Health Service (i.e., the dental or medical unit), while their use of the FSC varied. Some were former FCS clients who had previously used the service regularly but now only required psychiatric medication reviews a few times a year, while others were Aboriginal health workers who used FCS on an as needs basis (i.e., a few times a year if in crisis). A small number were family members of participating clients who had asked to participate in the study (i.e., they had found out about the study through the participating family member). Thirty nine per cent of participants (*n* = 32) elected to be interviewed in person, and 61 per cent (*n* = 49) elected to fill out the structured interview as a pen-and-paper questionnaire. Table 1 presents social and cultural demographic information of the participants.

**Table 1:** Participants demographics

	Total	Percentage
Number of participants	81	
Indigenous status		
Aboriginal	67	83%
Torres Strait Islander	0	0
Aboriginal and Torres Strait Islander	0	0
Bicultural (identified with Indigenous and non-indigenous heritage/family cultural backgrounds)	14	17%
Identified as Koori (born in Victoria)	68	84%
Non-Koori (born outside Victoria)	13	16%
Age (years), Mean (SD)	41 (SD 13)	
Sex		
Women	43	53%
Men	38	47%
Employment		
Yes	45	55%
No	36	45%
Financial security		
Enough money for basic living expenses		
Yes	49	60%
No	32	40%
Able to borrow $2000 within a week for something considered important, such as an emergency		
Yes	41	51%
No	40	49%
Secondary (year 12 high school) and higher education		
Completed secondary schooling	18	22%
Completed a diploma/certificate qualification (e.g., TAFE level)	55	70%
Completed a higher education tertiary degree	5	6%

## Measures

### *Australian Aboriginal version of the Harvard trauma questionnaire* [19]

The Australian Aboriginal Version of the Harvard Trauma Questionnaire (AAVHTQ) is a 47-item measure adapted from the Harvard Trauma Questionnaire, a cross-cultural trauma inventory developed at the Indochinese psychiatric clinic at Harvard University [34]. The AAVHTQ comprises two subscales designed to assess individual levels of trauma exposure and posttraumatic symptomology. The first subscale lists 17 potentially traumatic events. Respondents are asked if they have ever witnessed or experienced each of the 17 events, where they believed that they or someone else could have been killed or seriously harmed, or where they experienced feelings of intense helplessness, fear or horror when it happened. The second subscale of the AAVHTQ contains 30 items, 16 of which correspond to the PTSD symptom criteria in the Diagnostic and Statistical Manual of Mental Disorders III-R (DSM-III-R) [35], and 14 cultural idioms of distress, identified by C. Atkinson [19], that lie outside the DSM-III-TR PTSD criteria. The subscale is designed to produce two scores, a composite symptom severity score and a DSM-III-R PTSD total score. The first 16 items assess the extent to which respondents experience three symptom clusters associated with PTSD: re-experiencing of trauma events, physiological arousal, and avoidance and emotional numbing. The next 14 items assess the extent to which respondents experience a range of cultural idioms of distress, such as symptoms related to interpersonal difficulties (e.g., problems making or keeping relationship), suicidal thoughts, guilt and shame, dissociation, and alcohol and drug misuse. All 30 items are rated on 4-point Likert-scale (where 1 = not at all, 2 = a little bit, 3 = a fair bit, and 4 = a lot). C. Atkinson calculated the sensitivity and specificity of the AAVHTQ by examining the degree to which the threshold score of 75 from all 30 items of the AAVHTQ correctly classified participants as PTSD symptomatic (according to their mean scores on the 16 PTSD specific DSM-III-R items). The sensitivity and specificity of the AAVHTQ was 82.4%, and 98.5%, respectively [19].

### *Negative life events scale* [36]

The Negative Life Events Scale consists of 16 negative life events and asks respondents “Have any of these things been a worry for you or anyone else living in this house during the last year?” Participants are asked to simple ‘yes/no’ responses to the life events. The NLES has been shown to demonstrate adequate discriminant validity and internal consistency in two samples of Aboriginal participants living in remote communities of the Northern Territory, with Cronbach’s alpha levels reported to be 0.80 and 0.81 [37].

### The Aboriginal resilience and recovery questionnaire [30]

The Aboriginal Resilience and Recovery Questionnaire (ARRQ) is a 60-item multidimensional strengths questionnaire that was designed to assess strengths and resources associated with resilience, healing and recovery among Aboriginal and Torres Strait Islander help-seeking populations. The ARRQ use a 5-point Likert scale response format (1 = not at all, 2 = a little, 3 = somewhat, 4 = a fair bit, and 5 = a lot) and includes a wide range of resilience constructs such as community connection, community opportunity, cultural identity, self-worth, emotion regulation, positive emotions, strong relationships, safety, social support, a personal sense of mastery, spirituality as a source of strength, and participation in cultural practices. A principal components analysis suggested the retention of two component subscales, representing personal strengths and relational-cultural strengths, while internal consistency tests recommended by Funk [56] indicated sufficient psychometric properties for use of the ARRQ either as a composite total strengths score measure, or as the two component subscales. A reliability analysis revealed that both subscales and the total strength scores demonstrated high internal consistency, with a Cronbach’s alpha value of 0.89 for the personal strengths component, 0.88 for the relational-cultural strengths component, and 0.93 for the total strengths composite measure of the AARQ. Findings in this study used the total strength composite scores of the ARRQ. Pearson correlation coefficients with Aboriginal-designed measures of drug and alcohol use, empowerment and historical loss show that the ARRQ has demonstrated good convergent and discriminant validity [30].

### Recruitment and interview process

The recruitment and interview process has been described in full elsewhere [30]. In brief, Aboriginal help-seeking clients were recruited through use of a flyer in the FCS waiting room, and by FCS staff (e.g., counselors, psychiatrists, GPs). All staff involved in recruitment had reviewed the structured interview and safety processes, and many staff had helped design the research process. Staff were given a plain language statement for interested clients that outlined the project aims, time required for the interviews, and the availability of counselors post-interview. Information provided to client was explicit about the interview involving asking clients about the types of traumatic events they had experienced in their life. Fortnightly staff meetings ensured regular discussion about the project’s progress and any safety or recruitment issues that might arise. Inclusion criteria for recruiting clients included being over 18 years of age, and being mentally and emotionally well/stable enough to participate in a 60–90 min interview about trauma, resilience, healing and recovery, without experiencing a level of distress that would risk exacerbating any current difficulties or conditions. This safety measure excluded clients currently experiencing acute distress or severe mental health problems. Accordingly, only a limited number of clients attending the FCS were asked to participate in the project during 2011.

With prior consent from the respective client, participants were contacted by telephone or in person by the first author. Interviews were conducted in the service counseling rooms, most of the time just prior to the clients seeing their regular FCS counselor, drug and alcohol worker, GP or psychiatrist. Clients could choose to be interviewed in person or complete the measure as a pen-and-paper questionnaire. The average time for completing the structured interviews was 90 min (range 45–120 min). The debriefing time post-interview included providing refreshments, information for follow up support if participants experienced any distress post-interview, and an opportunity to provide feedback. Participants were given a 75 dollar shopping voucher upon completion of the interview as a gesture of appreciation for generously sharing their time and experiences.

## Results

### Data preparation and descriptive statistics

Univariate tests were conducted to examine assumptions of normality and homogeneity of variance for all variables, including visual inspection of a range of graphs and parametric and nonparametric assumptions tests. Initial inspection of the distribution of scores revealed that four participants in the sample reported scores on a majority of measures that were located in the extreme ranges. These participants were excluded from the analysis. Table 2 summarises the minimum, maximum, mean scores, standard deviations, and skewness and kurtosis statistics for each measure. The patterns of results indicate that assumptions of normality were met for the total strength ARRQ scores, and negative life events scores, whereas the distribution of trauma symptom scores exhibited significant kurtosis (kurtosis = -1.24, standard error 0.55).

**Table 2:** Descriptive statistics

Scale	N	Min	Max	Mean	SD	Skewness	SE	Kurtosis	SE
Trauma events	75	3.0	21.0	12.73	4.29	-.48	.28	-.32	.55
Trauma symptoms	75	3.0	87.0	35.58	23.71	.11	.28	-1.24	.59
Negative Life Events	72	1.0	15.0	6.13	3.42	.46	.28	-.59	.56
Total Strengths	73	118.0	235.0	180.74	28.85	-.15	.28	-.58	.56

### Trauma symptoms and PTSD classification

To determine participant’s PTSD symptomatic status, procedures outlined by C. Atkinson [19] were followed. C. Atkinson’s research work in developing the AAVHTQ did not involve clinician-rated diagnoses. Rather, based on recommendations from Mollica and colleagues [34], a mean score of 2.5 for the 16 DSM-III-R items were selected to classify participants as PTSD symptomatic or non-symptomatic. In this study, 40 per cent of the participants completing all thirty items of the AAVHTQ (*n* = 30/75) scored greater than or equal to 2.5, and were classified as PTSD symptomatic. Forty-eight per cent (*n* = 36/75) scored greater than or equal to 2.5 for the 16 DSM-III-R items. This results in a sensitivity of 83 per cent, and a specificity of 81 per cent for the AAVHTQ to correctly classify participants using the PTSD symptomatic threshold.

### Pearson correlations and independent samples t-tests

Pearson’s *r* correlations were used to investigate the relationships between trauma exposure, trauma symptom severity, racism and total strengths (resilience). Table 3 presents the correlations between measures used in this study. Results indicated a strong, positive correlation between trauma exposure and trauma symptom severity (*r* = 0.59, *p* < 0.01), and trauma symptom severity and negative life events (*r* = 0.57, *p* < 0.01), and a strong, negative correlation between total strengths resilience trauma symptom severity (*r* = -0.56, *p* < 0.01).

**Table 3:** Pearson correlations

Variable	1	2	3	4
Trauma exposure				
Trauma symptom severity	.59**			
Racism	.46**	.36**		
Negative life event	.30*	.57**	.23*	
Total Strengths	-.20	-.56**	-.24*	-.42**

* *p* < .05, ** *p* < .01

There was also a small, positive correlation between racism and trauma symptom severity (*r* = 0.36, *p* < 0.01), accounting for 12 percent of the variance in total trauma symptom severity. An independent t-test conducted to test whether participants with a personal history of child removal from natural family reported higher trauma symptom severity than those who were not removed indicated there was no difference in means scores between those reporting a history of child removal compared to those did not (*t* [72] = 1.56,* p* = 0.12). Post-hoc independent t-tests were conducted to investigate whether there was any difference in mean trauma symptom scores, and levels of trauma exposure, for participants with two generations of child removal (including personal removal and either one parent or a child having been removed from their natural family) compared to those who did not report two generation of removal in their family. Those participants reporting two generations of child removal reported higher trauma symptom severity (*M* = 47.33, *SD* = 18.92) compared to those who did not experience two generation of removal (*M* = 33.35, *SD* = 23.99), *t* [18] = 2.24,* p* = 0.038). In addition, those participants reporting two generations of child removal reported experiencing a higher number of traumatic events in their lives (*M* = 14.5, *SD* = 3.2) compared to those who did not experience two generations of removal (*M* = 12.01, *SD* = 4.5), *t* [72] = 2.26,* p* = 0.02). An independent t-test conducted to test possible differences in trauma symptom severity between men and women, revealed that men reported higher mean trauma symptom severity scores (M = 43.49, SD = 24.28) in comparison to women (M = 28.67, SD = 21.17), t (73) = 2.82, p < .01, despite there being no difference in levels of trauma exposure, t (73) = 1.60, p = .12. 

Pearson’s *r* correlations were used to explore relationships between trauma symptom severity and several factors from the ARRQ that are frequently cited as cultural determinants of wellbeing or factors integral to Aboriginal and Torres Strait Islander social and emotional wellbeing [57, 58] . There were large, medium and small negative correlations between trauma symptoms severity and safety (*r* =—0.51, *p* < 0.001), community opportunity (*r* =—0.38, *p* < 0.001), and communal mastery (*r* = -0.27, *p* < 0.01), whereas the associations between trauma symptoms severity and community connection (*r* =—0.23, *p* > 0.01), cultural identity (*r* =—0.09, *p* > 0.01), cultural practices (*r* = 0.09, *p* > 0.01), and spirituality (*r* = 0.05, *p* > 0.01), were non-significant.

### Hierarchical regression analyses

A hierarchical regression analysis was conducted to investigate the potential moderating effect of total strengths on the relationship between trauma exposure and trauma symptom severity, such that higher levels of total strengths would be associated with a significantly weaker relationship between trauma exposure and trauma symptom severity, after controlling for sex, education and access to basic living expenses. Moderation effects were assessed by examining the significance of appropriate interaction terms, and bootstrapping methods were used to assess both simple and multiple mediations using bias-corrected confidence intervals [38]. Table 4 presents the results of the regression analysis. There was a main effect for basic living expenses in step one of the equation, and main effects for both trauma exposure and total strengths in step two. In the third step of the equation, after controlling for trauma exposure and the total strengths component, the interaction of trauma exposure and total strengths component made a significant contribution to trauma symptoms. Hence, total strengths moderated the relationship between trauma exposure and trauma symptom severity.

**Table 4:** A hierarchical regression analysis testing the moderating effect of total strengths on the relationship between trauma exposure and trauma symptom severity

Variable	Β	*P*	Adj. R^2^
Step 1			
Sex	.19	.11	.20
Education	-.20	.09	
Basic expenses	-.30	.02*	
Step 2			
Sex	.11	.19	.57
Education	-.10	.25	
Basic expenses	-.13	.15	
Total strengths	-.40	.00**	
Trauma exposure	.44	.00**	
Step 3			
Sex	.12	.15	.59
Education	-.07	.41	
Basic expenses	-.11	.21	
Total strengths	-.44	.00**	
Trauma exposure	.49	.00**	
Total strengths x Trauma exposure	-.17	.049*	

^*^ *p* < .05, ^**^ *p* < .01

**Fig. 1:**
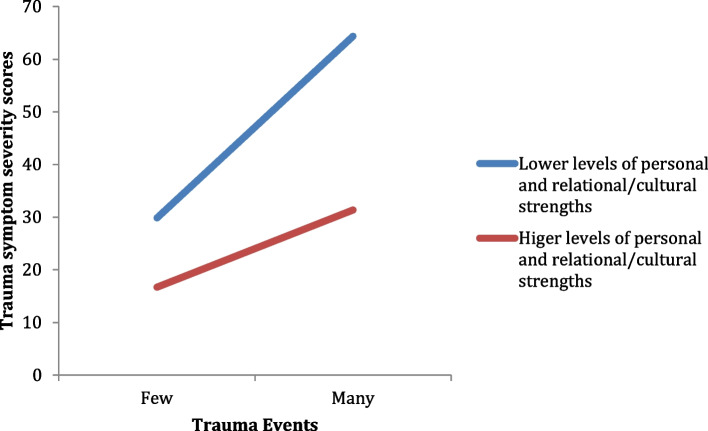
The moderating effect of total strengths on the relationship between trauma exposure and trauma symptom severity

To determine the nature of this moderation, the relationship between trauma events and trauma symptoms at low and high levels of total strengths (i.e., -1 SD and + 1 SD from the mean) were plotted. Figure 1 shows that the slope was different from zero for those with low levels of total strengths, *t* (69) = 9.24, *p* < 0.001, whereas it was not for those with high levels of total strengths, *t* (69) = 1.81, *p* < 0.074. Hence, at low levels of resilience the relationship between trauma exposure and trauma symptom severity is significant, whereas at high levels of resilience there is no longer a significant relationship between trauma exposure and trauma symptom severity.

An exploratory hierarchical regression analysis was conducted to examine the relative contribution of trauma exposure, negative life events, and total strengths, as predictors of trauma symptom severity, when controlling for gender, education lehierarchical regression analysis testing the contribution of trauma exposurevels, and access to basic living expenses. In the analysis, gender, education levels, and access to basic living expenses were entered in the first step of the analysis. Trauma exposure, hierarchical regression analysis testing the contribution of trauma exposure life event and total strengths scores were entered in the second step. Table 5 presents the results of this analysis. The main effect of basic living expenses accounted for 20 per cent of the variance in trauma symptom severity. On the second step the main effects for trauma exposure, negative life events, and total strengths, together accounted for an additional 40 per cent of the variance in trauma symptom severity.

**Table 5:** A hierarchical regression analysis testing the contribution of trauma exposure, negative life events and total strengths as predictors of trauma symptom severity

Variable	Β	*p*	Adj. R^2^
Step 1			
Sex	.19	.11	.20
Education	-.20	.09	
Basic expenses	-.30	.01*	
Step 2			
Sex	.09	.27	.60
Education	-.09	.28	
Basic expenses	-.13	.15	
Trauma exposure	.41	.00**	
Negative life events	.23	.03*	
Total strengths	-.34	.00**	

* *p* < .05, ** *p* < .01

## Discussion

The Indigenous literature on resilience, healing and recovery from trauma has identified common factors documented in the broader trauma recovery literature (e.g., establishing safety, constructing meaning from traumatic events), in addition to cultural determinants of wellbeing (e.g., cultural identity, cultural practices) and collective processes of healing (e.g., strengthening self-determination, building community capacity, restoring cultural norms). The majority of literature is qualitative and this makes it difficult to establish the extent to which these risk and protective factors differentially contribute to trauma-related outcomes. The aims and findings of this study were part of a larger research program undertaken that examined historical loss, contemporary trauma, and resilience and recovery outcomes among Aboriginal help-seeking clients using an Aboriginal community controlled counselling service [30]. In the present study we investigated associations between several unexplored, culturally salient factors – specifically, client’s experiences of child removal from natural family, racism, and being male—and posttraumatic stress symptom severity. We also investigated the potential moderating role of resilience (i.e., client’s access to strengths and resources, including cultural strengths) and the contributions of trauma exposure, stressful life events, and resilience in predicting trauma symptom severity.

Before discussing these results, we note several broader findings with regards to the assessment of trauma exposure and trauma symptom severity. First, on average, participants reported experiencing a high number of traumatic events during their lifetime (mean = 12.73). Notably, when we compare this finding with those from studies using the original Harvard Trauma Questionnaire with marginalised cultural groups, the extent of trauma exposure in this study was comparable to that reported in studies examining refugee populations who have experienced large-scale collective trauma [39]. The elevated rates of trauma exposure in this study are consistent with large scale studies involving American Indian populations that found a risk of higher rates of trauma exposure for both men and women in two American Indian reservation communities [40]. We also noted that 40 per cent of the participants reported experiencing symptom patterns consistent with PTSD when assessed using the Aboriginal Australia Version of the Harvard Trauma Questionnaire. Further, similar to Atkinson’s findings, participants reported experiencing a broad range of symptoms of distress that included trauma and culturally salient idioms of distress [19]. This is an important finding for practitioners and services because it reinforces results from studies indicating the importance of assessing all three trauma related domains of distress when working with Aboriginal and Torres Strait Islander people.

Most participants in this study reported having experienced racial discrimination in their lives, and there was a significant association between the extent of racism experienced and posttraumatic stress symptom severity. This is consistent with survey findings that among Aboriginal and Torres Strait Islander people racism is a stressor associated with higher rates of psychological distress, substance abuse, and poorer self-assessed general health and mental health [41]. The finding is also consistent with research conducted among other minority groups that have found a relationship between experiences of racism and PTSD [42]. For example, Matheson and colleagues (2019) conducted surveys with First Nation Canadian, Metis and Inuit adults recruited from community/health centres in Canada and found that after controlling for education, posttraumatic stress symptom severity was associated with greater perceived discrimination [43].

Posttraumatic stress symptom severity among participants with a history of childhood removal did not differ to those with no such history. However, a post-hoc analysis revealed that participants who reported two generations of child removal in their families, reported experiencing more traumatic events in their lives and higher trauma symptom severity, in comparison to those who did not. This finding supports the view that multiple generations of child removal in families elevates the risk of experiencing adversity and confer a vulnerability to experiencing mental health and social and emotional wellbeing difficulties [44]. The finding that male participants reported higher trauma symptom severity in comparison to than female participants, despite experiencing no differences in levels of trauma exposure, is contrary to worldwide epidemiological data that has consistently found higher prevalence rates of PTSD and higher levels of trauma exposure among females [45]. However, with respect to gender and trauma exposure, Beals and colleagues also reported comparable levels of trauma exposure between men and women in a large study involving nearly 2000 adults from two American Indian reservation communities [40]. The finding is also congruent, with the focus group discussions that preceded this study, in which Aboriginal health staff at the counselling service observed that males appeared to be particularly vulnerable to the impact of historical trauma and the loss of land, language and identity [30]. Intergenerational trauma theories described by J. Atkinson [46] and Miller [47] proposed that the loss of traditional knowledge, roles and status for some Aboriginal males as a result of colonisation leads to poor self-image and uncertainty about the future. This, when combined with poor coping skills and elevated rates of substance use, can manifest as frustration and aggression, potentially escalating into violence and other stressful life experiences.

There was a strong, negative association between participants’ composite total strengths scores on the Aboriginal Resilience and Recovery Questionnaire [30] and trauma symptom severity, with total strengths contributing a substantive 31 per cent of the variance in trauma symptom severity. The subscales of the ARRQ include a range of personal strengths (e.g., self-worth, emotional regulation), relationship strengths (e.g., access role models, social support), community strengths (e.g., opportunities in community, communal mastery) and cultural strengths and resources (e.g., cultural identity). As such, the finding is consistent with investigations of models of resilience as a multidimensional construct that includes personal, social and environmental capacities [48, 49]. Significantly, we found that total strength scores of the AARQ moderated the relationship between trauma exposure and trauma symptom severity. Specifically, the regression model showed that the relationship between trauma exposure and trauma symptom severity was no longer significant when high levels of resilience were entered into the model. Whilst this study is cross-sectional and cannot establish a causal directionality, this moderation finding is consistent with the idea that the capacity of clients to access and draw upon strengths and resources is a factor in mitigating posttraumatic stress symptom severity.

It highlights the importance of practitioners taking a strength-based approach to therapeutic work with Aboriginal and Torres Strait Islander help-seeking clients who have experienced significant trauma. This finding contributes to the emerging evidence base documenting the moderating effects of resilience in the area of mental health and wellbeing [50, 51]. One other study finding a moderating effect of resilience specific to interpersonal trauma exposure (i.e., not combat exposure) and trauma symptom severity, was conducted by Fincham and colleagues [52]. They found that resilience moderated the relationship between childhood abuse and PTSD symptoms among 787 secondary school students from five public secondary schools in Cape Town, South Africa. Our finding is also consistent with Indigenous intergenerational trauma theories that emphasise the importance of culture, relationships and community connectedness in healing trauma [25, 28, 53, 54].

We investigated the contributing roles of resilience, trauma exposure and stressful life events together when predicting trauma symptom severity, while controlling for access to basic living expenses. It is noteworthy that all three factors, in addition to access to basic living expenses, all uniquely predicted trauma symptom severity, accounting for 60 per cent of the variance in trauma symptom severity. Within the context of supporting Aboriginal and Torres Strait Islander help-seeking clients with histories of trauma, the finding highlights the need for services to take an interdisciplinary approach that includes social work, case management and other relevant services that can help clients to address these three areas of wellbeing. That is, support clients to establish a level of financial security, mitigate the impacts of stressful life events (including exposure to interpersonal trauma, such as experiences of racism, family and community violence), and strengthen access to personal, relationship, community and cultural resources (i.e., resilience).

Several important limitations with regards to the findings need to be noted. First, the study was cross-sectional and therefore unable to determine individual post trauma trajectories and causal directions. Hence, participants reporting low trauma symptoms and high strengths could have experienced resistance (e.g., no trauma impact), resilience (e.g., early trauma impact followed by a fast return to baseline functioning) or recovery (e.g., slow return to baseline functioning) post trauma pathways. The study design also means it is not possible to determine whether the strengths outlined in the ARRQ played an antecedent role in contributing to post-trauma outcomes. It is possible that participants reporting high levels of posttraumatic stress symptom severity may currently view themselves in a more negative light and underestimate their current strengths, and/or that over time, the impact of posttraumatic stress has contributed to depleting these strength-related psychosocial and cultural resources. Another limitation of the study is that it is not clear how generalisable the findings are to other Aboriginal and Torres Strait Islander population groups. The study is limited on two fronts with regards to this issue. First, the clients recruited were a non-probability sample and not representative of the whole counselling service clinical population. For safety and ethical reasons the inclusion criteria for participation precluded those clients that were either currently suffering acute distress, or experiencing long-term severe mental health problems such that the interview could potentially exacerbate any current conditions. In addition to not being a non-probability sample, the participants from the study cannot be viewed as a ‘clinical population’ in that a proportion of participants were also former counselling clients that now used the service on an as needs basis, while others were users of the broader health service or family members of participants. The service site for this research was small, and this level of flexibility for participation was required in order to make the study feasible. Second, and more broadly, due to the paucity of current research in this area, it is difficult to compare these findings with other Aboriginal and Torres Strait Islander communities and cultural groups. The findings may have differed significantly had the research been conducted in more remote or rural Aboriginal and Torres Strait Islander communities.

Finally, a limitation of this study lies in our use of a PTSD symptom structure that is now outdated and has since undergone multiple revisions. The Australian Aboriginal Version of the Harvard Trauma Questionnaire [19] is the only current culturally adapted PTSD measure available, and it was developed using PTSD symptom criteria from the Diagnostic and Statistical Manual of Mental Disorders III-R [35]. Whilst efforts are underway to develop an Aboriginal and Torres Strait Islander designed PTSD measure that includes the most recent PTSD symptom clusters [20] these initiatives had not yet commenced at the time of this study and that research is still in progress. We also note that current evidence suggests the most recent PTSD factor structure may not represent the best symptom structure among non-Western populations [55].

These limitations notwithstanding, our findings demonstrated that a range of personal, relationship, community and cultural strengths were found to be associated with lower posttraumatic stress outcomes, and to have a greater protective effect at higher levels of trauma exposure. In addition, several historical and culturally salient factors were found to be predictors of trauma symptom severity. These findings add to the Aboriginal and Torres Strait Islander, and broader Indigenous trauma and recovery literature .

## Data Availability

The data that support the findings of this study are available from the Victorian Aboriginal Health Service but restrictions apply to the availability of these data, and so are not publicly available. Deidentified data are available from the corresponding first author (GG) upon reasonable request and with permission of the Victorian Aboriginal Health Service.

## Footnotes

1 This article uses the term the term ‘Indigenous’ and ‘First Peoples’ for broad reference to First Peoples worldwide and ‘Aboriginal and Torres Strait Islander to respectfully refer to the diversity of First Peoples in Australia. Specific terms for different First Peoples when citing relevant literature is referenced accordingly. These terms are used deliberately to reflect the preferences held by many Aboriginal and Torres Strait Islander peoples and other respective Indigenous First Peoples worldwide.

2 The term ‘*Koori*’ (meaning ‘people’ in Eastern and Western Kulin dialect) is used to refer specifically to any Aboriginal person or peoples that identify as belonging to one or more of the traditional Aboriginal language or clan groups located in Victoria, Australia. The term ‘Aboriginal Victorians’ will be used more broadly to refer to all Aboriginal peoples living in Victoria.
